# Short-term information processing, long-term responses: Insights by mathematical modeling of signal transduction: Early activation dynamics of key signaling mediators can be predictive for cell fate decisions

**DOI:** 10.1002/bies.201100172

**Published:** 2012-04-23

**Authors:** Annette Schneider, Ursula Klingmüller, Marcel Schilling

**Affiliations:** Division Systems Biology of Signal Transduction, DKFZ-ZMBH Alliance, German Cancer Research Center (DKFZ)Heidelberg, Germany

**Keywords:** cell fate decision, gene regulatory network, integrated response, mathematical model, signaling dynamics

## Abstract

How do cells interpret information from their environment and translate it into specific cell fate decisions? We propose that cell fate is already encoded in early signaling events and thus can be predicted from defined signal properties. Specifically, we hypothesize that the time integral of activated key signaling molecules can be correlated to cellular behavior such as proliferation or differentiation. The identification of these decisive key signal mediators and their connection to cell fate is facilitated by mathematical modeling. A possible mechanistic linkage between signaling dynamics and cellular function is the directed control of gene regulatory networks by defined signals. Targeted experiments in combination with mathematical modeling can increase our understanding of how cells process information and realize distinct cell fates.

## Introduction

The transmission and processing of information is an essential element of multicellular organisms to ensure the coordinated function of different cells and cell types. Fundamental cell fate decisions such as differentiation, proliferation, and programmed cell death (apoptosis) are tightly regulated by signals that cells receive from their environment. These signals are processed by complex intracellular signaling networks to finally result in distinct cellular behavior.

The encoding of the input information into post-translational modifications of signaling molecules and the subsequent decoding into defined outputs is a highly nonlinear process. It involves multiple steps and regulation mechanisms including positive and negative feedback loops as well as crosstalk between different signal inputs. An increasingly employed strategy to examine these complex relations is mathematical modeling, facilitating the simultaneous analysis of various signaling events 1, 2. Understanding cellular information processing can help gain insights into the cause of uncontrolled signaling in diseases such as cancer and to develop improved treatment regimes. Even though complete comprehension of cell fate decisions remains challenging, progress is being made to unravel the cellular signaling code.

### Short-term signaling determines long-term cell fate

One approach to dissect cellular decision making is to link distinct signaling patterns with cell fate. However, whereas the initial activation of cellular signaling occurs within minutes to hours, the adaptation to a certain cellular behavior frequently involves profound structural changes within the cells that require a timeframe of hours to days. It is unclear at what time point an individual cell is committed to a specific cell fate.

A first step to get insight into the mechanism of cell fate decision is the identification of correlations between processes related to information processing and cell behavior. If such correlations exist, then it is likely that the underlying mechanisms are also connected physically. Already in the 1980s it was reported that processes at the receptor level can predict cell proliferation in response to epidermal growth factor (EGF): mathematical modeling revealed that there is a linear relationship between proliferation and the number of EGF receptor-ligand complexes at steady state 3. Later, refined receptor models including growth factor binding and internalization were used to analyze strategies to control cell proliferation 4. Similarly, mathematical models of the trafficking dynamics of the IL-2 and IL-4 receptor could predict T cell proliferation 5, 6.

A question that remained open was how events at the receptor level are processed in the cell. An important discovery was the notion that, in contrast to most of the receptor studies mentioned above, it is the dynamics of intracellular signal transduction rather than the steady state that is the key mechanism to encode information 7. A famous example is the finding that rat adrenal medulla cells react differently depending on the duration of extracellular signal-regulated kinase (ERK) activation: sustained ERK activation that is induced by stimulation with nerve growth factor (NGF) leads to neurite outgrowth, whereas transient ERK activation following EGF stimulation results in proliferation 8. A connection between signaling dynamics and receptor occupancy was made by a study analyzing T cell activation as a function of ligand bound T cell receptors: T cells with a low number of stimulated T cell receptors showed a transient intracellular Ca^2+^ signal and failed to be activated, but when a certain threshold of occupied T cell receptors was reached, the intracellular Ca^2+^ signal was sustained and the cells were activated to secrete IFN-γ 9. Further evidence for the importance of the temporal dynamics of signaling pathways was provided recently. Several groups have combined quantitative dynamic data with mathematical modeling, strongly suggesting that the dynamics of the initial signaling response can be predictive for cell fate decisions.

One example is the reaction of erythroid progenitor cells to the cytokine erythropoietin (Epo). These cells require small amounts of Epo to survive, whereas high Epo concentrations initiate cell proliferation. This behavior is reflected in intracellular signaling patterns. Low Epo concentrations are sufficient to activate STAT5, and the amount of phosphorylated STAT5 in the nucleus during the first hour after Epo treatment was shown to directly correlate with survival of erythroid progenitor cells 10. High Epo concentrations additionally result in the phosphorylation of ERK. Congruently, the amount of activated ERK during the first hour could be linked to the proliferation degree of erythroid progenitor cells 11. Both studies benefited from mathematical modeling to solve the problem that physiological responses, such as survival, can already be triggered by stimulus at concentrations that are very low and not sufficient to elicit an experimentally measurable signaling response. Only with the help of model simulations was it possible to connect the measured signaling dynamics with cell fate decisions that were measured at much lower stimulus concentrations. An important prerequisite for performing such predictions is to establish a model that is able to describe the dose dependency of the signaling pathway 12.

Similarly, in a human cancer cell line treated with the pro-apoptotic cytokine TNF-α in combination with the pro-survival factors EGF or insulin, apoptotic responses have been linked to short-term signaling. Using partial least-squares regression analysis it was demonstrated that signaling events up to 90 minutes after receptor activation correlated best with apoptosis-survival decisions 13. This suggested that signaling activities at early times encode much of the information needed to specify life or death decision. Another study analyzing apoptosis mediated by TNF-related apoptosis-inducing ligand (TRAIL) showed that the time between TRAIL exposure and apoptosis is highly variable. However, this cell-to-cell variability could be attributed mostly to the rate of conversion of the BID protein to the truncated version tBID by the enzyme caspase-8 14. This knowledge allowed the time to cell death in single cells to be accurately predicted by following caspase activity over time.

Finally, particular signaling patterns were connected to migration and proliferation in the context of cancer development. In growth factor-treated breast cancer cells with different oncogene expression levels, partial least-squares regression analysis identified quantitative combinations of signals occurring between 5 and 30 minutes that strongly correlated with cell proliferation and migration 15.

In summary, with the help of mathematical modeling several studies could directly correlate the dynamics of early signaling with long-term cellular responses. This suggests that cell fate is determined long before it is apparent in cellular behavior.

### Cellular responses can be decoded by focusing on the integral of key mediators

The dynamics of a typical intracellular signal are defined by several properties: the time of the maximum signaling peak, the duration of the signal and the peak amplitude 16. Which of these features determines a specific cell fate and could therefore be employed to predict the cellular response beforehand?

**Figure 1:**
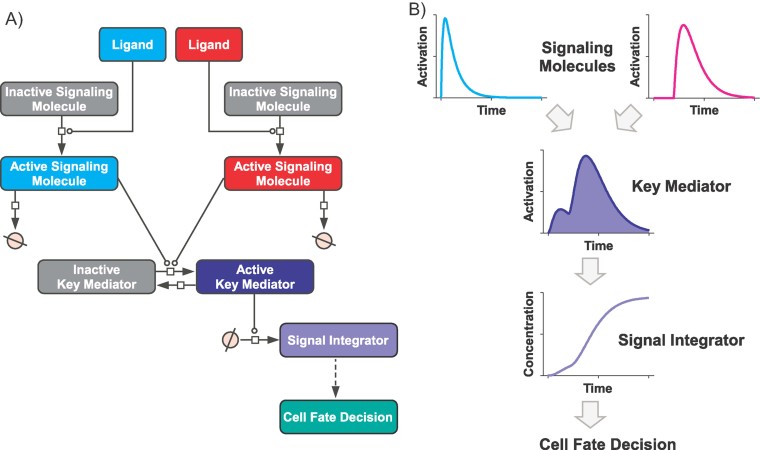
The integrated activation curve of a key mediator can predict cell fate. **A:** Scheme of an exemplary signaling pathway, in which stimulation with two different ligands leads to activation of distinct signaling molecules. This information is summarized by a key mediator protein and processed by a signal integrator, resulting in a specific cell fate. Lines with open circles: reaction catalysis; crossed circle: unspecified source or sink. Layout according to Systems Biology Graphical Notation 39. **B:** Activation dynamics of the signaling molecules shown in (A). The two different ligands stimulate different signaling molecules both of which can activate the same downstream molecule. Since this molecule summarizes the information from both upstream signaling molecules and transfers it to another molecule, it functions as a key mediator. The integral of the activated key mediator, defined as the area under the signaling curve, is illustrated by the filled area. This quantity directly correlates with the dynamic behavior of a long-living signal integrator and is thus a decoder for the information from the input signals. Potential signal integrators are stable mRNA molecules and the proteins they encode, which then execute the specific cell fate.

We propose that a signal property that comprises both the kinetics and the magnitude of the signal can be linked to the physiological output of the cell: the integral of the signaling curve. This quantity represents the integrated signaling response over time (Fig. 1) and can be calculated by computing the area under the curve, with the signal at time point 0 treated as offset. The integral describes how many signaling molecules are active over a certain time. Provided that the output behavior is slow compared to the signaling process, the output is an integrator of the upstream signal and measures how long and with which intensity the signal has been on 17. Since the accomplishment of cell fate decisions is a slow process, using the integral of the signaling curve to predict cell fate is reasonable. For theoretical studies such as sensitivity analysis using the integral as a variable to define signaling dynamics is already a standard method 18.

In the studies mentioned above that analyzed the responses of erythroid progenitor cells to Epo, it was the integral of activated STAT5 and ERK, respectively, that could be correlated to survival and proliferation 10, 11. In line with this result, the integrated ERK activity induced by fibronectin was previously shown to be proportional to DNA synthesis in CHO cells 19. Additionally, the analysis of cytokine-induced apoptosis in colon adenocarcinoma cells revealed that signaling could only be linked to the apoptotic response when derived metrics were used for the model predictions, such as the integral or steady state levels of key proteins 13. To explain this, the authors hypothesized that the critical signaling information is embedded in the cumulative activities and rates of change of key signals rather than in the ordering of signaling events.

These studies pointed towards a correlation between specific signal properties and cell fate. How can we elucidate the underlying mechanism? To reveal the mechanistic link between cause and effect it is not sufficient to collect more data, since with this approach we will only find more correlations. Instead, we could try to reduce the problem by identifying which properties of the signaling kinetics are really important. To answer this question, the system has to be challenged by targeted perturbations to specifically affect a certain signal property. For instance, it was demonstrated that the amplitude of a signal depends on the activity of kinases, whereas the duration of a signal depends on phosphatases 16. These signal properties might be specifically altered by overexpression or inhibition of the kinases and/or phosphatases involved. Mathematical modeling could support the identification of conditions, allowing for optimal discrimination between different scenarios. Connecting the different signaling kinetics with physiological readouts could then validate the hypothesis that the integrated response of a specific signal can determine cell fate. This knowledge could be a basis to find the mechanistic connection between signal integration and cellular behavior.

A related open question is the integration time of the signal. Theoretically, this corresponds to the time interval during which the area under the curve is to be computed. In practical terms, this represents the time by which the cell is committed to its long-term fate. In the last paragraph, we discuss possible molecular mechanisms to set this threshold.

### Distinct key mediators process specific cellular information

Another crucial issue is to choose the right key signal that is directly correlated to cell fate. A signaling network consists of many reactions and certainly a specific cell fate is dependent on many of them 20. However, it should be possible to identify key mediator proteins such as ERK that summarize the information of the several upstream signaling pathways and thus determine the cell's response to input signals 21. Mathematical modeling facilitates the identification of key mediators, and complex signaling networks can be reduced to much simpler computational models that are directly connected to biological outcomes 22.

**Figure 2:**
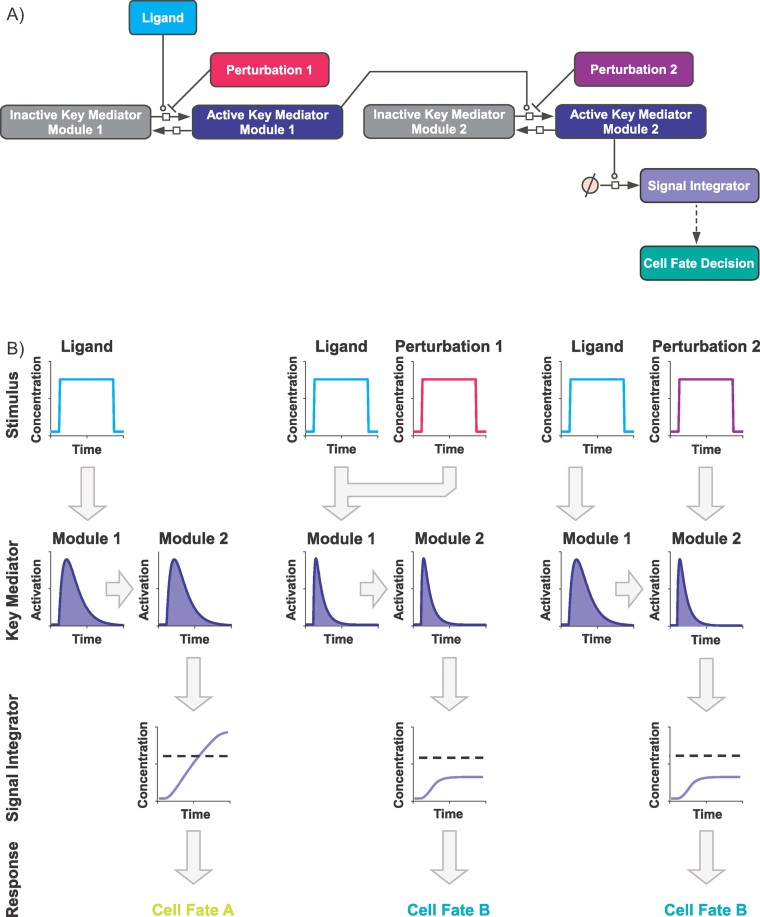
The definition of a key mediator can be dependent on the stimulation condition. **A:** Example of a network structure that is separated into different modules and processes information dependent on the signaling input. Lines with open circles: reaction catalysis; lines with perpendicular bars: reaction inhibition; crossed circle: unspecified source or sink. Layout according to Systems Biology Graphical Notation 39. **B:** Integration of different inputs in different modules for the network shown in (A). In the left panel, a specific ligand leads to the activation of a key mediator that is the output of module 1. Since there is no perturbation downstream of module 1, the integral of the key mediator is directly proportional to the concentration of the signal integrator. The cell might decide for cell fate A if, for example, the concentration of the signal integrator exceeds a certain threshold. In the middle panel, the ligand is combined with a perturbation, which might be a second ligand or a specific inhibitor of a signaling molecule. This combination results in a decreased integral of the key mediator summarizing module 1, and consequently to the decision for cell fate B. Thus, submodule 1 is still predictive for cell fate. This is not the case for the situation depicted in the right panel. Here, the costimulation does not alter the integral of the key mediator of module 1. On the other hand, cell fate is no longer correlated to module 1. Hence, the perturbation takes places downstream of module 1 and cell fate decision is dependent on module 2.

The situation is even more complex when a cell is subjected to diverse stimuli. Proteins that have been identified to be a key mediator for a certain stimulus are not necessarily a key mediator for a combination of different stimuli. For instance, following TNF-α stimulation early Akt signaling was related to anti-apoptotic decisions, but only when the cells lacked a functional TGF-α autocrine circuit 12. Similarly, in adenovirus-infected cells treated with a combination of IFN-γ and TNF-α, activated Akt was predictive for apoptosis, but not when PI3-kinase was inhibited 23. These examples show that it is crucial to critically evaluate the correct key signaling mediator for each stimulus condition. Nevertheless, the excitation of a system with different input signals is a useful method to gain insight into the wiring of the network. An approach to disentangle the network structure, and thus to identify a key mediator for a certain condition, is the separation of the signaling network into defined modules. These modules are connected, for example, by crosstalk, but may be analyzed independently from each other 24. Even if two pathways operate in parallel and are interlinked via crosstalk, mathematical modeling can help to modularize this network into smaller entities. As a simplification, the output of one module can then be considered as the input to the next module (Fig. 2A) 25.

If a signaling molecule that was identified as the key mediator under a certain stimulus or stimulus combination (Fig. 2B, left and middle panel) does not correlate with cell fate once another stimulus is present, then the second stimulus probably affects a module downstream or parallel of the first stimulus (Fig. 2B, right panel). As mentioned above, the integral of activated ERK is linked to DNA synthesis and proliferation, but this is only the case if downstream or parallel modules are not perturbed. For instance, in the study analyzing proliferation in response to fibronectin, the integral of ERK activation was no longer proportional to DNA synthesis if the cells were additionally stimulated with insulin. This demonstrated that the signaling crosstalk underlying the response synergism did not converge at ERK activation. Instead, the signaling molecule IRS-1 was shown to be activated both by fibronectin and insulin and was suggested to be a point of signaling crosstalk 19.

The principle can be further illustrated by the following example: growth factors stimulate proliferation by inducing cyclin D expression via activated ERK. If cyclin D levels exceed a certain threshold, leading to hyperphosphorylation of the Rb protein, cell fate is committed to DNA synthesis and cell division. This point of no return is referred to as the restriction point of cell cycle 26. However, anti-proliferative signaling through TGF-β triggers the inhibition of Rb phosphorylation, and in this way counteracts cell division 21, 27. Thus, under these conditions, it is no longer the ERK module that is predictive for cell fate, but rather the Rb module that is further downstream.

Simultaneous stimulations with different factors can reveal whether a second signal or perturbation affects the predictive power of a defined key mediator. If a second stimulus interferes at a level upstream of, or parallel to, the key mediator, it alters the dynamic behavior of the key mediator. If the cellular response changes in the same direction, the key mediator is still connected to cell fate (Fig. 2B, middle panel). Based on this, successive stimulations could answer the question of whether there is a critical time window after which the second stimulus no longer influences cell fate because enough activated key mediators have been accumulated by this stage to direct the cell into a specific behavior.

### The black box: The connection between signaling and cell behavior

Little is known about how a distinct cell behavior is eventually established after a signal has been received. Certainly, for most processes synthesis of new proteins that fulfill regulatory or structural function is required. The timely expression of these proteins can be ensured by organized transcriptional networks, commonly referred to as gene regulatory networks (GRN). In GRNs the interaction of distinct transcription factors results in the establishment of a specific transcriptional state, maintained by repression of some genes and activation of others. For instance, in developmental biology GRNs are analyzed to understand the commitment to a specific cell type, and several motifs controlling individual subcircuits in GNRs have been described 28, 29. Similar to the situation in signal transduction, the analysis of the properties of different regulatory motifs and how they interact is facilitated by mathematical modeling 29, 30. However, although our understanding of signaling on the one side and gene regulation on the other is increasing, little is known about the connection of the two. How does the establishment or stabilization of a specific state in a GRN depend on the dynamics of signal transduction? And finally, how is the coordinated expression of selected genes transferred into a specific cellular function? We have taken the first steps in answering these questions by identifying the correlative nature of certain processes, but how they are mechanistically connected still remains to be clarified.

**Figure 3:**
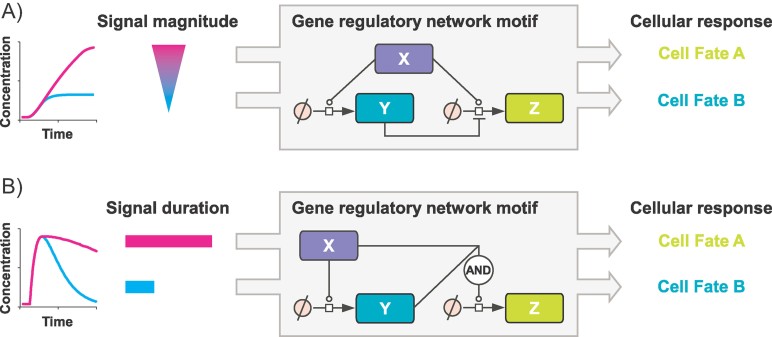
Possible mechanisms for decoding the information from the integrated signaling response. **A:** Signal magnitude: in the depicted incoherent feed forward loop motif the active protein X activates both Y and Z, but Y inhibits Z. Dependent on the amount of X and the activation sensitivity of either Y or Z, the network stabilizes in favor of Y or Z, and thus for cell fate A or B. Since the integral of the signal determines the amount of X, it can be directly correlated to cell fate. **B:** Signal duration: in the coherent feed forward loop, the first activated molecule X not only activates Y, but also supports its function to induce Z. Dependent on the temporal availability of X, only Y is activated or additionally Z, which can be translated to cell fate A or B. Lines with empty circles: reaction catalysis; lines with perpendicular bars: reaction inhibition; crossed circle: unspecified source or sink. Layout according to Systems Biology Graphical Notation 39.

Nevertheless, progress is being made in connecting signaling properties with transcriptional networks. As mentioned before, we speculate that the integral of an activated key mediator is connected to the fate of the cell. In general, this information has to be decoded by one or several signaling integrators. Likely candidates for these integrators are mRNAs and their protein products that might control distinct subcircuits of a GRN and thus execute a specific cell fate. Ultimately, the signal integrator might be manifested in the network dynamics that in turn are dictated by the structure of the GRN. This would imply that the whole network constitutes the integrator, not single molecules. Based on recent studies, we propose two possible mechanisms that might be responsible for the decoding of the integrated signaling response. Basically, they interpret two properties that comprise the integral: the magnitude and the duration of a signal (Fig. 3).

First, if the signal results in the expression of a stable transcription factor, then the integral is proportional to the concentration of this transcription factor. The successive accumulation of a particular set of transcription factors could change the balance in a GRN that has more than one metastable state, thereby stabilizing one or other of the possible states 31. Indeed, the signal-mediated increase of a transcription factor was recently linked to a GRN executing a binary choice between two cell fates 32. Specifically, the strength of B cell receptor (BCR) signaling was connected to the decision of B cells to either change the immunoglobulin (Ig) repertoire or develop further into an antibody-secreting plasma cell. High versus low signal intensities evoked by different antigen affinities resulted in high versus low levels of the transcription factor IRF4. A combination of experimental data and mathematical modeling demonstrated that these differing levels of IRF4 decide B cell differentiation by controlling the duration of an obligate transcriptional state promoting Ig diversification: high IRF4 levels shortened this state and favored fast transmission to the plasma cell state. Thus, IRF4 served as a sensor of BCR signaling strength, translating it into a binary cell fate decision 32. The underlying network motif of this example is an incoherent feed forward loop, in which an activated protein X activates both Y and Z, but Y inhibits Z (Fig. 3A). Mathematical analyses recently demonstrated that an incoherent feed forward loop is able to decode fold changes of the signal input, which then determines the amplitude and duration of the response 33. Since the integral is the fold change computed over time, it is reasonable that it can be decoded by a comparable network structure.

A second possible mechanism to compute an accumulating or steady signal over time could be the constant requirement for an activated transcription factor to stabilize the transcriptional program or to progress through several stages to finally reach a stable condition. In this case, the underlying network motif could be a coherent feed forward loop, in which the first activated molecule X not only activates Y, but also supports its function to induce Z 34 (Fig. 3B). An example for this feed forward mechanism is the response to bone morphogenic protein (BMP) in the *Drosophila* embryo. BMP induces the expression of the transcription factor Zen via the latent transcription factor Smad. Only if BMP signaling continues, can Zen act together with Smad to induce another gene, Race 35. By combining experiments and mathematical modeling, a similar mechanism was described to discriminate between transient and sustained ERK activation via specific c-Fos expression 36. Another study in which a coherent feed forward might play a role specifically identified the integral of a signal as being crucial for determining positional identity in the neural tube 37. In neural progenitor cells, a gradient of the morphogen Shh controls the expression of a set of transcription factors, resulting in the diversification into different transcriptional domains and consequently cell types 38. Dessaud et al. 37 showed that it is actually not merely the concentration of Shh determining cell fate, but the cumulative level and duration of Shh signaling, and thus the integral of the signaling response. The authors proposed a feed forward model in which early activation of some genes alters the transcriptional state of the cell. This change in turn is a pre-condition for the induction of other genes occurring only in case of sustained Shh stimulation. Thus, the positional identity of a cell is determined by a dynamic mechanism, combining the ligand gradient and the state of the transcriptional network in the responding cell 37.

**Abbreviations and definitions**

**Akt:** Protein kinase with multiple isoforms that has pro-survival activity. Synonym for protein kinase B (PKB).

**BCR (B cell receptor):** Immunoglobulin that is expressed as antigen receptor on the cell surface of individual B cell clones. The recognition of its cognate antigen triggers the secretion of mature antibodies.

**BID (BH3-interacting domain death agonist):** The cleavage product of BID, tBID, enables the release of cytochrome c from the mitochondria, which triggers apoptosis.

**BMP (bone morphogenic protein):** Secreted proteins belonging to the TGF-β superfamily that play a role in embryonic development. Can operate as morphogen.

**Crosstalk:** The concept that signaling proteins of one pathway influence the proteins of another pathway. Crosstalk can take place between signaling networks activated by different receptors as well as between different pathways within a receptor network.

**Cyclin D:** One of the major proteins regulating cell cycle progression. Associates with cyclin-dependent kinases, causing their activation. Activated kinases phosphorylate Rb protein and thereby promote cell cycle progression.

**EGF (epidermal growth factor):** Growth factor that stimulates proliferation of various epidermal and epithelial cells.

**Epo (erythropoietin):** Major cytokine regulating erythrocyte differentiation and the maintenance of a physiological level of circulating erythrocytes.

**ERK (extracellular signal-regulated kinase):** Kinase with multiple isoforms activated by growth factors and involved in the regulation of cell proliferation. Belongs to the MAP (mitogen-activated protein) kinases that sequentially activate each other.

**Feed forward loop, coherent:** Network motif in which a regulator X regulates Y and Z, and Y also regulates Z. The direct regulation of Z by X has the same sign as the indirect regulation via Y.

**Feed forward loop, incoherent:** Network motif in which a regulator X regulates Y and Z, and Y also regulates Z. The direct regulation of Z by X has the opposite sign as the indirect regulation via Y.

**GRN (gene regulatory network):** A transcriptional network established by the reciprocal regulation of interacting genes.

**IFN-γ (interferon gamma):** Cytokine with antiviral and immunomodulatory function.

**IL (interleukin):** Large class of cytokines mainly playing a role in the immune system.

**Insulin:** Hormone involved in regulating blood glucose levels.

**Integral of the signaling curve:** The area under the activation curve of a signaling protein, corresponding to the integral of the activation function versus time.

**IRF4 (interferon regulatory factor 4):** One of the nine transcription factors in the IRF family playing a role in immune cells.

**Mathematical modeling:** Analysis of biological networks by mathematical descriptions, such as ordinary differential equations. After the parameters of the mathematical models are calibrated with quantitative biological data, the models are analyzed and employed for simulations and predictions.

**Morphogen:** Secreted protein that by its gradual distribution conveys positional information during embryonic development.

**NGF (nerve growth factor):** Growth factor involved in the development and maintenance of the sympathetic and sensory nervous systems.

**Partial least-squares regression analysis:** A statistical strategy to connect a matrix of variables (X) to a matrix of responses (Y). Used to find the vector in X that explains the maximum variance in Y in linear regression methods.

**PI3-kinase (phosphoinositide 3-kinase):** Kinase with multiple isoforms that consists of two subunits. Is involved in the regulation of cell growth, metabolism and proliferation. Phosphorylates phosphoinositide residues of cell membrane lipids, which subsequently serve as docking sites for Akt and many other signaling proteins.

**Rb (retinoblastoma protein):** Cell cycle inhibitor and tumor suppressor. Functions by inhibiting transcription factors that are necessary for cell cycle progression. Is inactivated by hyperphosphorylation (see cyclin D).

**Sensitivity analysis:** Mathematical analysis to investigate relative changes of derived system quantities as a result of relative infinitesimal changes in parameter values. It reveals which parameters have a large impact on the system.

**Shh (sonic hedgehog):** Secreted protein that plays a role in the development of the vertebrate central nervous system. Can operate as morphogen.

**STAT (signal transducer and activator of transcription):** Latent transcription factors mainly activated by cytokine receptors. In their phosphorylated form STAT proteins translocate to the nucleus and directly activate gene expression.

**TGF-α (transforming growth factor alpha):** A member of the EGF family that can activate the EGF receptor. Initiates cellular proliferation and migration, for example, in development and wound healing.

**TGF-β (transforming growth factor beta):** Growth factor regulating many cellular functions including cell growth, adhesion, migration, differentiation, and apoptosis.

**TNF-α (tumor necrosis factor alpha):** Cytokine binding to specific death receptors. Depending on the context, it can stimulate apoptosis, proliferation, or induce an inflammatory response.

**TRAIL (TNF-related apoptosis-inducing ligand):** Cytokine binding to a specific death receptor and thereby inducing apoptosis.

To sum up, a correlation of signaling dynamics and cell fate response is frequently observed, but how these processes are mechanistically connected still remains to be clarified. Nonetheless, first insights into the regulatory networks determining gene expression downstream of signal transduction provide some hints on how the dynamics of signaling and the organized expression of particular genes might be connected. Mathematical modeling can contribute to our understanding of how different signaling and gene regulatory motifs are linked.

## Conclusions

Cellular information processing is crucial for the integration of the signals that cells receive. Growing evidence supports our hypothesis that information is processed fast, with a timeframe of minutes to hours. This allows cells to rapidly react to changes in their environment. The response to these changes, represented by different cell fates, is accomplished in hours to days, and in most cases includes de novo gene transcription as well as structural changes within the cell. The combination of quantitative experimentation and mathematical modeling has demonstrated that it is possible to identify key mediators of signal processing. We propose that the integrated activation curve of these key mediators is frequently correlated with the cell's response to a signal. This might be achieved by a dynamic control of the GRN, resulting in the orchestrated expression of proteins that are necessary to execute a specific cellular behavior. The principle of key mediators will allow us not only to predict cell fate decisions before they are apparent, but also to gain a basic understanding about how a cell commits to a certain cell fate. This knowledge has direct implications for the rational design of new therapeutics to combat cancer and other diseases associated with deregulated cell fate decisions.
